# Gene Regulation in *Giardia lambia* Involves a Putative MicroRNA Derived from a Small Nucleolar RNA

**DOI:** 10.1371/journal.pntd.0001338

**Published:** 2011-10-18

**Authors:** Wei Li, Ashesh A. Saraiya, Ching C. Wang

**Affiliations:** Department of Pharmaceutical Chemistry, University of California San Francisco, San Francisco, California, United States of America; Jawaharlal Nehru University, India

## Abstract

Two core microRNA (miRNA) pathway proteins, Dicer and Argonaute, are found in *Giardia lamblia*, a deeply branching parasitic protozoan. There are, however, no apparent homologues of Drosha or Exportin5 in the genome. Here, we report a 26 nucleotide (nt) RNA derived from a 106 nt Box C/D snoRNA, GlsR2. This small RNA, designated miR5, localizes to the 3′ end of GlsR2 and has a 75 nt hairpin precursor. GlsR2 is processed by the Dicer from *Giardia* (GlDcr) and generated miR5. Immunoprecipitation of the Argonaute from *Giardia* (GlAgo) brought down miR5. When a *Renilla* Luciferase transcript with a 26 nt miR5 antisense sequence at the 3′-untranslated region (3′ UTR) was introduced into *Giardia* trophozoites, Luciferase expression was reduced ∼25% when synthetic miR5 was also introduced. The Luciferase mRNA level remained, however, unchanged, suggesting translation repression by miR5. This inhibition was fully reversed by introducing also a 2′-O-methylated antisense inhibitor of miR5, suggesting that miR5 acts by interacting specifically with the antisense sequence in the mRNA. A partial antisense knock down of GlDcr or GlAgo in *Giardia* indicated that the former is needed for miR5 biogenesis whereas the latter is required for miR5-mediated translational repression. Potential targets for miR5 with canonical seed sequences were predicted bioinformatically near the stop codon of *Giardia* mRNAs. Four out of the 21 most likely targets were tested in the Luciferase reporter assay. miR5 was found to inhibit Luciferase expression (∼20%) of transcripts carrying these potential target sites, indicating that snoRNA-derived miRNA can regulate the expression of multiple genes in *Giardia*.

## Author Summary

*Giardia lambia* is a deeply branched parasitic protozoan and the pathogen causing the diarrhetic disorder, giardiasis. The mechanism of gene regulation in this organism is largely unknown. Here, we identified a 26 nucleotide (nt) small RNA from the 3′-end of a 106 nt small nucleolar RNA (GlsR2) in *Giardia*. GlsR2 is processed through the action of a Dicer protein in *Giardia* to generate the 26 nt RNA. The latter becomes associated with the Argonaute protein. The protein-RNA complex can repress the translation of messenger RNAs carrying the antisense sequence of the 26 nt RNA at the 3′-untranslated region. This small RNA, designated microRNA5 (miR5), has several potential targets identified in *Giardia*, among which four were further tested in *Giardia* and found their translation repressed by miR5. This is the second functioning microRNA we have indentified in *Giardia*. The microRNAs could be thus important regulators of gene expression in this ancient single cellular organism.

## Introduction

The intestinal parasitic protozoan *Giardia lamblia*, one of the earliest branching eukaryotes, is the causative pathogen of a diarrheal disease giardiasis throughout the world. It has been included as part of the WHO Neglected Disease Initiative since 2004 [1]. Though the transcripts in *Giardia* are produced in the nucleus and transported to the cytoplasm for translation like the other eukaryotes, few consensus promoters have been identified. Most of the transcription factors identified in other eukaryotes are missing in *Giardia*. There is but one highly divergent TATA-binding protein [2]. An AT-rich region commonly located near the beginning of open reading frames is believed to function as the promoter [3]. The transcripts in *Giardia* have exceedingly short 5′-untranslated regions (UTRs) ranging mostly from 0 to 14 nucleotides and similarly short 3′-UTRs of 10 to 30 nucleotides [3]. This has ruled out some mechanisms of translational regulation, which are essential in higher eukaryotes, such as ribosome scanning in translation initiation [4]. Moreover, the machinery of RNA interference (RNAi) is absent from *Giardia*. A specific double-stranded (ds)RNA virus (Giardiavirus) with a 6,277 basepairs (bp) has been found actively multiplying in the cell [5], and long dsRNAs were not degraded in *Giardia* [6]. Apparently, foreign dsRNA cannot be degraded in *Giardia*.

microRNAs (miRNAs) are an ancient class of small RNAs that mediate post-transcriptional regulation of mRNAs in animals and plants, which is critical for many biological processes [7]–[9]. Functional 18∼24-nucleotide (nt) mature miRNAs are derived from primary transcripts (pri-miRNAs) that are usually several kilobases long non-coding RNAs [9]–[11]. Cleavage at the stem of a hairpin structure in the pri-miRNA by the nuclear RNase III Drosha releases a smaller hairpin structure, the precursor miRNA (pre-miRNA) [9], [11], which is exported to the cytoplasm by Exportin5, a member of the nuclear transport receptor family [12]. In the cytoplasm, pre-miRNAs are cleaved near the terminal loop by Dicer, releasing a ∼22 nt double-stranded (ds) miRNA intermediate bearing a 2 nt overhang at the 3′-end [13], [14]. This duplex, often imperfectly paired, is then associated with an Argonaute protein plus other proteins to release the antisense strand and assemble into a protein-RNA complex, RISC (RNA-induced silencing complex) [15], [16]. The complex binds to a partially complementary sequence in the 3′- UTR of the target mRNA and represses its translation [7], [8].

Homologues of Drosha or Exportin5 have not been found in *Giardia*, but a single functional Dicer (GlDcr) and a single Argonaute (GlAgo) homologue have been identified in the *Giardia* genome database [17], [18]. The crystal structure of GlDcr was recently resolved, representing the first Dicer structure available [17]. It consists of two coupled RNase III domains associated with a canonical PAZ domain. But it lacks the N-terminal DExD/H helicase, C-terminal double-stranded RNA binding domain and a DUF283 domain identified in the Dicers of higher eukaryotes [17]. The distance between the PAZ and the processing center predicts a small RNA product of ∼25 nt. GlDcr was shown to cleave short dsRNA (155 bp) *in vitro* to generate 25∼27 nt RNA and support RNAi in a *Schizosaccharomyces pombe* Dicer deletion mutant [17]. In view of the inconclusive indications on whether a small RNA-mediated posttranscriptional regulation could be operational in *Giardia*, we isolated, cloned and sequenced some of the small RNAs in the size range of 20∼30 nts from *Giardia* and identified one of the 26 nt small RNAs, miR2, as a Dicer-digested product from the 3′-end of GlsR17, an orphan snoRNA containing a C/D box without an antisense sequence to ribosomal RNAs (rRNAs) [18], [19]. A conserved putative target site for miR2 was identified at the 3′-UTRs of 22 variant surface protein (VSP) mRNAs. Expression of a reporter mRNA in *Giardia* carrying this putative target site at the 3′-UTR was repressed by miR2 without altering the mRNA level. This repression, which was dependent on the presence of GlAgo, indicated the ability of this snoRNA-derived small RNA to function as a miRNA in RISC-mediated translational repression in *Giardia* [18].

In order to expand this observation to see if more functional miRNAs are derived from the snoRNAs in this organism [19], we pursued the identification and characterization of a new *Giardia* miRNA, miR5, from the 3′ end of another *Giardia* box C/D snoRNA, GlsR2. The result provided extensive evidence for the presence of another functional snoRNA-derived miRNA in *Giardia*.

## Materials and Methods

### Cell culture

*Giardia lamblia* (WB clone C6, ATCC50803) trophozoites were cultured as described previously [20]. Cells were grown anaerobically in plastic culture tubes at 37°C in the modified TYI-S-33 medium supplemented with antibiotics.

### RNA isolation

Total RNA was isolated from *Giardia* trophozoites using Trizol (Invitrogen), while size-fractioned small RNAs (<200 nts) were isolated using the *mir*Vana kit (Ambion).

### Northern blot analysis

MAXIscript Kit (Ambion) was used to incorporate α-^32^P-UTP (Perkin Elmer) into the RNA probes. DNA probes for RL, RL-TS (see below), and 16S rRNA were generated from PCR using γ-^32^P-ATP (Perkin Elmer) end-labeled gene specific primers. Fifteen µg of size-fractionated small RNAs (<200 nts) were separated in a 12% denaturing polyacrylamide gels (8 M Urea, 1× TBE (Tris-Borate-EDTA) buffer) and capillary blotted onto a Hybond-N membrane (Amersham) followed by UV light irradiation. Blots were hybridized with the radiolabeled probes overnight at 42°C in a solution containing 50% formamide, 0.5% SDS, 5xSSC (150 mM NaCl, 15 mM sodium citrate), 5xDenhardt's solution and 100 µg/ml denatured salmon sperm DNA. The blots were washed twice with 2xSSC and 0.1% SDS for 15 min at room temperature followed by two washes with 0.1xSSC and 0.1% SDS for 15 min at 42°C. The hybridization signal was monitored with a PhosphorImager screen and scanned with a GE Storm 860 (Amersham).

### Primer extension

Primer Extension System–AMV Reverse Transcriptase (Promega) was used for primer extension.

A cDNA sequencing ladder was obtained using *fmol* DNA Cycle Sequencing System (Promega). Primer (5′-GGC TCG GAC ATC CAA GG-3′) (IDT) used for cDNA sequencing and primer extension was PAGE-purified and γ-^32^P-ATP (Perkin Elmer) end-labeled using T4 polynucleotide kinase (New England Biolab). The cDNA thus synthesized was analyzed by electrophoresis in 8% polyacrymide/8 M urea gel along with the GlsR2 cDNA sequencing ladder. The gel was exposed to a PhosphorImager and scanned with GE Storm 860 (Amersham).

### *Giardia* small RNA cloning and 3′-end sequencing

*Giardia* small RNAs (20∼30 nts) were cloned as described previously [18]. The RT-PCR products thus derived were used as the template for PCR aimed at amplifying the 3′ end of miR5. The products were then cloned into pGEM-T Easy vector using the pGEM-T Easy kit (Promega). *Escherichia coli* colonies containing the inserts were collected and the plasmid DNA was isolated and sequenced.

### GlDcr immunoprecipitation and *in vitro* dicing assay

The N-terminal tagged (3×c-myc tags) GlDcr was integrated into the plasmid pNlop4 and expressed *in Giardia* cells as described [21]. For immunoprecipitation of GlDcr, 10 µl of anti-c-myc beads (Pierce) were incubated with 1% BSA for 12 hrs at 4°C, and then incubated with *Giardia* cell lysate of 5×10^7^ cells in 0.5% BSA for 12 hrs at 4°C. The beads were extensively washed with Tris buffered saline (TBS), suspended in 50 µl Dicer storage buffer (50 mM Tris-HCl, pH 7.6, 50 mM NaCl, 5 mM MgCl_2_, 20% glycerol) and stored at −20°C. For *in vitro* dicing assay, 2 µl of the beads was incubated at 37°C with 500 ng of a substrate in the presence of 3 mM MgCl_2_, 30 mM NaCl and 100 mM Hepes, pH 7.5. *In vitro* transcribed GlsR2 (106 nts) and pre-miR5 (75 nts) were each trace-labeled using the MAXIscript Kit (Ambion). The final volume of each reaction was 10 µl. Reactions were stopped by adding 10 µl of formamide gel loading buffer. RNA fragments were resolved by denaturing polyacrylamide (12%) gel electrophoresis and visualized by phosphor imaging. The same reactions and electrophoresis were carried out using unlabeled *in vitro* transcribed GlsR2 and pre-miR5. The digested products were transferred onto a Hybond-N membrane (Amersham). An anti-miR5 sequense (5′ AAG GCT CGG ACA TCC AAG GAA GCA TC 3′) was γ-^32^P-ATP (Perkin Elmer) end-labeled and used as the probe in a Northern.

### GlAgo immunoprecipitation and detection of miR5 by RT-qPCR

The N-terminal 3-HA tagged GlAgo was immunoprecipitated as described [21]. For RT-qPCR of miR5, the extracted ∼26 nt RNA band co-immunoprecipitated with GlAgo was extracted from the gel and reverse transcribed using the SuperScript III RT (Invitrogen) with the RT primer 5′ GTC GTA TCC AGT GCA GGG TCC GAG GTA TTC GCA CTG GAT ACG ACA AGG CTC G 3′. miR5 cDNA was then amplified using iQ Supermix (Bio-Rad), with a forward primer 5′ ACG ATG CTT CCT TGG ATG TC 3′, a reverse primer 5′ TAT CCA GTG CAG GGT CCG A 3′ and a TaqMan probe 5′ CTG GAT ACG ACA AGG CTC GGA CA 3′. The PCR products were analyzed by 2% agarose gel electrophoresis.

### Assay for miR5 function

*Giardia* WB strain wild-type cells, GlAgo-knockdown cells or GlDcr-knockdown cells [18] were grown in modified TYI-S-33 media to a density of 10^7^ per ml, washed twice in phosphate buffered saline (PBS), once in electroporation buffer (10 mM K_2_HPO_4_–KH_2_PO_4_ (pH 7.6), 25 mM HEPES (free acid), 120 mM KCl, 0.15 mM CaCl_2_, 2 mM EGTA, 5 mM MgCl_2_, 2 mM ATP, 4 mM glutathione), and then suspended in the electroporation buffer. Capped mRNA (4 µg), yeast tRNA (125 µg), synthetic 5′-phosphate-miR5 RNA (miR5, 1 µg, IDT) or synthetic 2′-O-methylated antisense of miR5 (ASO-miR5, 1 µg, IDT) were added in various combinations to the cell suspension, incubated on ice for 10 min and subjected to electroporation using a Bio-Rad Gene Pulser Xcell (Voltage: 450 V, Capacitance: 500 mF, Resistance: ∞). Cells were then incubated on ice for 10 min, added to pre-warmed culture medium, incubated at 37°C for 4 hrs, pelleted, washed once in PBS, and lysed using the *Renilla* Luciferase assay kit (Promega). The lysate was centrifuged at 12,000×g for 2 min to remove cellular debris. The cleared lysate was used to assay for *Renilla* Luciferase activity. The protein concentration of the cleared lysate was measured by the Bradford method (Bio-Rad) and used to normalize the Luciferase activity.

## Results

### GlsR2 can be processed to a 26 nt RNA

The first and only functional miRNA found in *Giardia* thus far, miR2, was a 26 nt small RNA derived from an orphan box C/D snoRNA GlsR17 [18]. GlsR2 is another box C/D snoRNA identified among the cDNA clones of a *Giardia* small RNA library [19]. The cloned GlsR2 cDNA has an 11 nt antisense element in the 5′-domain and was postulated to guide the 2′-O-methylation of Cm334 in the 16S rRNA of *Giardia*. Primer extension of the 16S rRNA, however, failed to show methylation at position C334 and the real function of GlsR2 in *Giardia* remains unclear [19].

**Figure 1:**
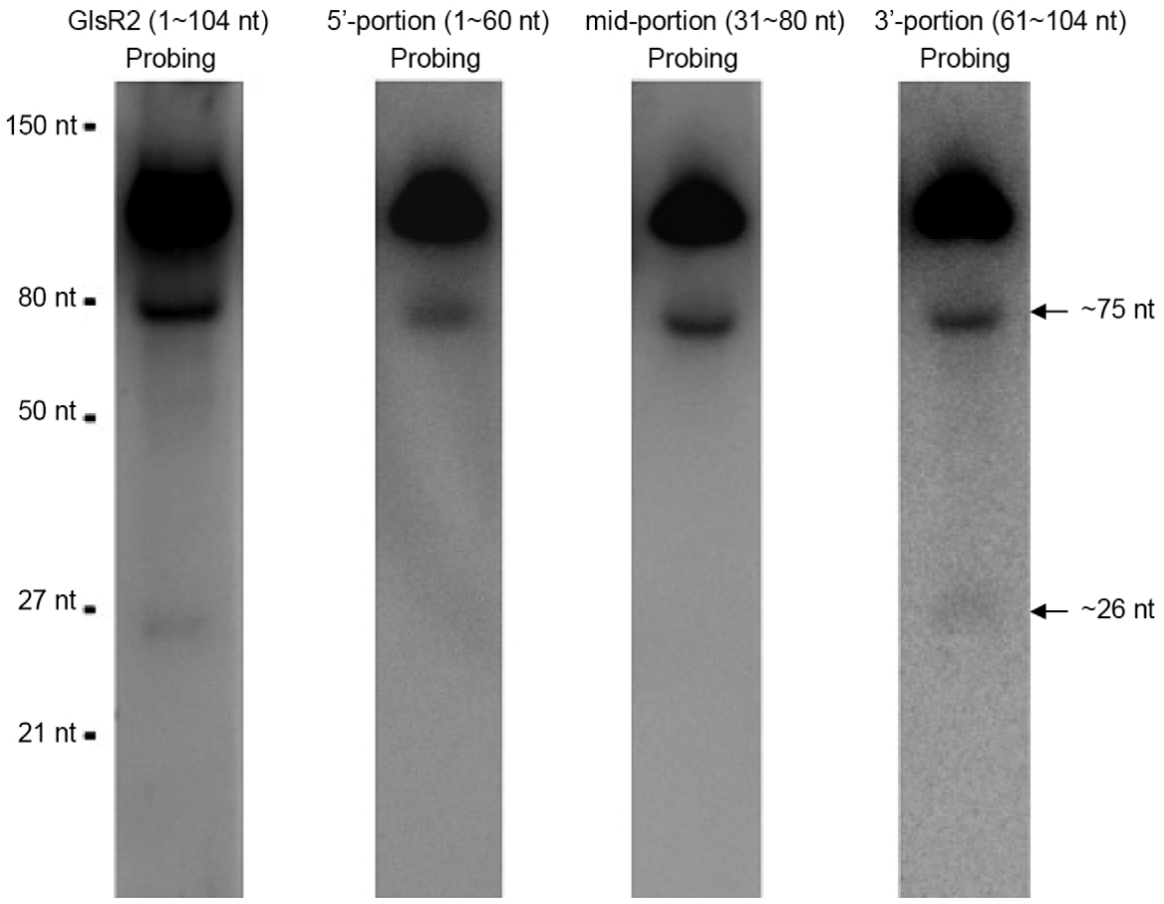
GlsR2 can be processed to a small RNA of 26 nts. Northern blot analysis of GlsR2 using *in vitro* transcribed antisense RNA of full-length, 5′-portion, mid-portion or 3′-portion of GlsR2 as probe. Fifteen micrograms of size-fractionated small RNAs (<200 nts) were used for each blot. The probe used for the blot is shown on top of each lane.

To see if GlsR2 could be processed to a smaller RNA species in *Giardia*, Northern blot assays were performed using 15 µg of size-fractionated small RNAs (<200 nts) probed with the full-length GlsR2 antisense RNA (Figure 1). The results showed that a band with an estimated size of about 75 nt and a very faint band of about 26 nt could be detected in addition to the full length GlsR2 band. To determine which part of GlsR2 may have generated the two smaller RNA species, the sequence of GlsR2 cDNA was divided into three overlapping portions; the 5′-portion (1∼60 nt), the mid-portion (31∼80 nt) and the 3′-portion (61∼104 nt) (Figure 1), and their antisense RNAs were used as probes in Northern blots. All three probes were capable of hybridizing to the full-length GlsR2 band (Figure 1). The 75 nt band was intensely stained by the mid-portion probe and the 3′-portion probe but only weakly hybidized with the 5′-portion probe. Only the 3′-portion probe was able to detect the 26 nt RNA band. Thus, the 26 nt RNA is probably derived from the 3′ portion of GlsR2.

### Identification of the precise 5′ and 3′ ends of the GlsR2-derived RNAs

**Figure 2:**
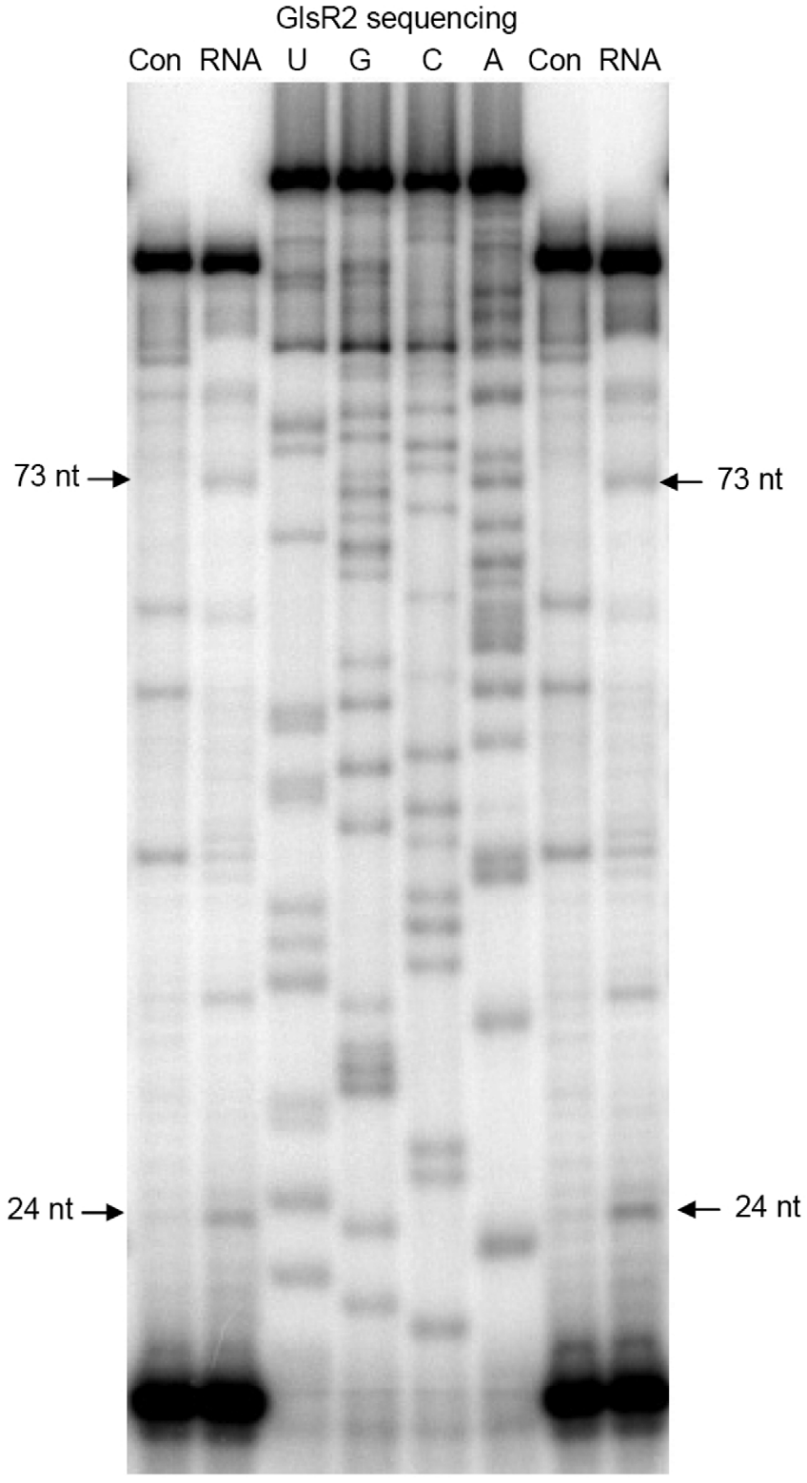
Determination the 5′ end of GlsR2-derived small RNA by primer extension. A 17 nt end-labeled primer complementary to the 3′ end of GlsR2 were used in the primer extension reactions. *In vitro* transcribed GlsR2 (0.75 ng) was used as a control template (Con) to show the secondary structure stops. Size-fractioned small RNA (<200 nts, 4 µg) was used as another template (RNA) to identify the mature miR5 and its potential intermediary precursor. GlsR2 sequencing reactions, using the same primer, were run along with the primer extension to determine the sequences of the products.

To determine the precise 5′ ends of both the 75 nt RNA and the 26 nt RNA located at the 3′ portion of GlsR2, primer extension assays were performed (Figure 2). A 17 nt end-labeled primer complementary to the 3′ end of GlsR2 [19] was used. Four µg of size-fractionated small RNAs (<200 nts) was used for the primer extension. An *in vitro* transcribed GlsR2 (0.75 ng) was used as a control template in order to distinguish nonspecific stops caused by the secondary structures of GlsR2 from the real stop. GlsR2 sequencing reactions were run in parallel to identify the precise stopping sites of the primer extension products. Figure 2 shows the products from two separate primer extension reactions run in the same gel along with the GlsR2 sequencing ladder. Two bands of 24 nt and 73 nt were identified in the two primer extension reactions but were absent from the control lanes. The 24 nt band could correspond to the ∼26 nt RNA band whereas the 73 nt band could appear as the ∼75 nt band in the Northern (see Figure 1 and below). From the flanking sequence ladders, the 5′-end of the 24 nt RNA was positively identified to be G81, whereas that of the 73 nt RNA was A32.

The detection of a 73 nt and a 24 nt RNA fragment by primer extension instead of the 75 nt and 26 nt pieces seen in the Northern (Figure 1) was puzzling. The 3′-ends of the primer extension products were defined by the primer, which was synthesized according to the published sequence of GlsR2, with a defined full-length of 104 nt [19]. We decided to re-examine the 3′-ends of GlsR2 and the two smaller RNA species derived from it. A 20 nt primer complementary to the 3′-linker and a 20 nt primer starting from the 5′ end (G81) of the 24 nt small RNA defined by the previous primer extension were used to amplify a *Giardia* small RNA cDNA library. The PCR products were cloned and sequenced. All the sequencing results from the 12 isolated clones showed an additional pair of UU added to the reported GlsR2 3′-terminus. The UU comes from the genomic sequence. Apparently, a slight degradation happened at the 3′ end of GlsR2 when it was originally cloned and sequenced by Yang *et al.* [19], causing the loss of the UU pair. The full length of GlsR2 is thus most likely 106 nts.

**Figure 3:**
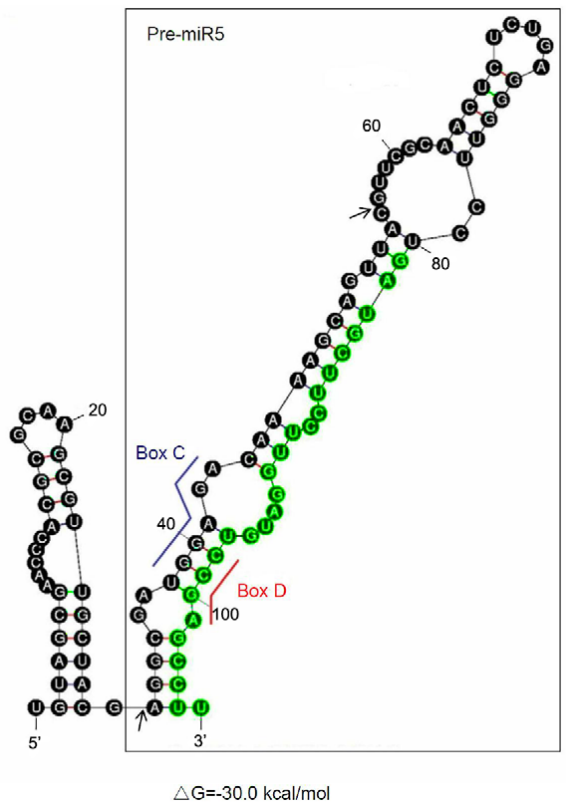
MFOLD predicted structure of snoRNA GlsR2. The sequence of the full-length GlsR2 was submitted to the MFOLD RNA server. Putative box C and box D of GlsR2 are marked by the blue and red line, respectively. The 75 nt miR5 precursor, pre-miR5, folds into a hairpin structure suitable for Dicer cleavage (black boxed). miR5 is located in the stem region of the hairpin at the 3′ end and colored in green. The arrows showed the 5′ and 3′ ends of the 25 nt small RNA discovered from deep sequencing library which could be miR5*.

Thus, the sequence of GlsR2-derived small RNA is 26 nts and defined as: 5′- GAU GCU UCC UUG GAU GUC CGA GCC UU -3′. It was subsequently designated as miR5 due to its biological activity (see below). Its precursor (pre-miR5) is most likely the 75 nt RNA which starts with A32 and ends with the UU pair. In our recent deep sequencing data for GlAgo-associated small RNAs, there are 1121 hits for the 26 nt miR5 sequence and 107 hits for the 25 nt sequence from the 5′ end of pre-miR5 (5′-AGG CGA UGG AGA CAA AAG CAG UUA C-3′), which forms double strand RNA with miR5 in the hairpin structure (Figure 3). The latter could be thus the miRNA*, which is probably also incorporated into Ago and regulate the expression of target genes [9], [11]. This miR5* has a 2 nt overhang at the 3′-end, suggesting the specific feature from GlDcr processing. No apparent iso-miR5 sequence was detected in the deep sequencing data.

The secondary structures of GlsR2 were analyzed by MFOLD (Figure 3). The putative box C (nts 38–43) and box D (nts 98–101) form a stem suitable for binding to the snoRNPs. The 75 nt putative precursor of miR5 (pre-miR5) is folded into a hairpin structure, which could serve as a substrate of GlDcr (Figure 3, black boxed). The enzyme for converting GlsR2 to the 75 nt pre-miR5 remains to be further verified (see below).

### GlDcr is required for processing GlsR2

**Figure 4:**
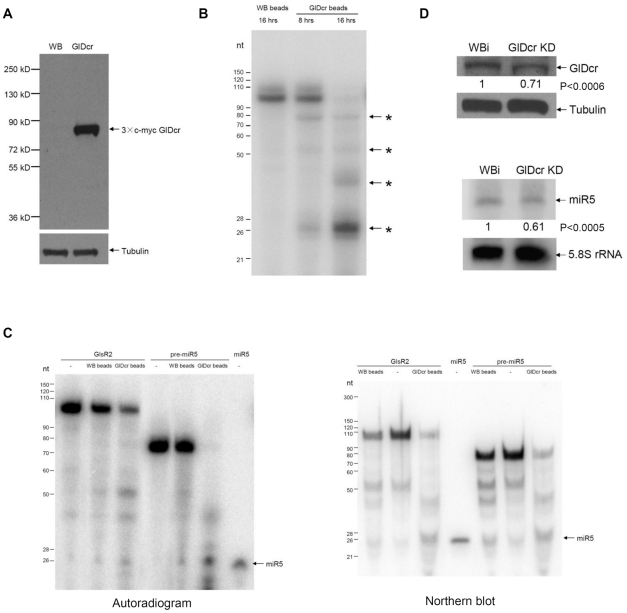
GlDcr is required for processing GlsR2 to generate miR5. (**A**) Western blot of 3×c-myc GlDcr that was immunoprecipitated using the anti-c-myc antibody. GlDcr: 3×c-myc GlDcr over-expressing cells. WB: un-transfected cells. Tubulin: loading control. (**B**) *In vitro* dicing of a 106 bp ^32^P-dsRNA. The labeled dsRNA was incubated with the immunoprecipitate from the untransfected cells (WB beads, left lane) and the transfected cells (GlDcr beads, center and right lanes) and incubated at 37°C for 8 or 16 hrs. The smaller RNA fragments were shown with asterisks. (**C**) *In vitro* dicing of GlsR2 and the 75 nt pre-miR5. Left panel: The purified GlDcr beads were incubated with the radiolabeled substrates at 37°C for 16 hours and analyzed with RNA PAGE and autoradiography. *In vitro* transcribed GlsR2 and pre-miR5 incubated with water (-) and WB beads were included as controls. Right panel: Unlabeled GlsR2 and pre-miR5 were digested by the GlDcr beads. After RNA PAGE, the gel was blotted and analyzed by a Northern using end-labeled anti-miR5 as probe. WB beads and water (-) were also included as the controls. (**D**) GlDcr is essential for biogenesis of miR5. Upper panel: Western blot using anti-GlDcr polyclonal antibody showed that GlDcr was knocked down by 29% in GlDcr knockdown (GlDcr KD) cells. WBi was the control cells and Tubulin was the loading control. Lower panel: Northern blot shows that the endogenous miR5 level was decreased by 39% in GlDcr KD cells. The 5.8S rRNA was used as a loading control. The results were from three independent experiments. The p-values indicated were calculated by two-tailed Student's *t*-test.

N terminal tagged GlDcr (3×c-myc GlDcr) was over-expressed in *Giardia*, pulled down by anti-c-myc beads, washed thoroughly (Figure 4A), and used in the *in vitro* dicing assay. GlDcr was shown to cleave short dsRNA (155 bp) to generate 25∼27 nt RNA *in vitro* [17]. A ^32^P-labeled double stranded (ds) RNA (106 bp) was used as a substrate to test the tagged-GlDcr beads and was found to be processed to smaller RNA fragments with a 25∼27 nt RNA band as the primary product (Figure 4B). A time course of the processing was followed at 8 and 16 hrs of incubation, and the results showed that 16 hrs are required for a thorough digestion of the substrate (Figure 4B). We then tested the GlDcr beads on ^32^P-labeled full-length GlsR2 or pre-miR5 and both were processed to a 26 nt RNA band, suggesting that both RNA species could be processed by GlDcr to produce miR5 (Figure 4C, left panel). To confirm that the 26 nt RNA is the miR5 itself, we repeated the same experiments with unlabeled GlsR2 and pre-miR5 and monitored the products with an anti-miR5 sequence in a Northern analysis. The result, presented in the right panel of Figure 4C, indicates that miR5 is a product from GlDcr digestion of either GlsR2 or pre-miR5. There is, however, no apparent conversion from GlsR2 to pre-miR5, suggesting that this particular reaction could be catalyzed by an enzyme other than GlDcr. Other apparent RNA bands were also found among the products from GlDcr-beads digestion as well as the control (Figure 4C). They could be derived from some potential minor contaminating RNases in *Giardia* not fully washed off the beads.

Additionally, we analyzed the biogenesis of GlsR2 in a GlDcr knockdown strain of *Giardia* trophozoites. GlDcr-antisense-hammerhead ribozyme RNA was used to partially knock down the expression of GlDcr [18]. Western blot showed that GlDcr protein was decreased by 29% in GlDcr knockdown cells comparing with the control (Figure 4D, upper panel). Size-fractionated small RNAs (<200 nts) were extracted from both the GlDcr knockdown cells and the control cells and examined for miR5 in a Northern blot. The results indicate that miR5 is decreased by 39% in the GlDcr knockdown cells (Figure 4D, lower panel). The study was repeated three times with similar outcomes, which underwent statistical analysis and resulted in a p value<0.0006 for the reduction in GlDcr and <0.0005 for the corresponding drop in miR5. It is thus likely that GlDcr is required for *in vivo* biogenesis of miR5.

### miR5 is associated with GlAgo in *Giardia*

**Figure 5:**
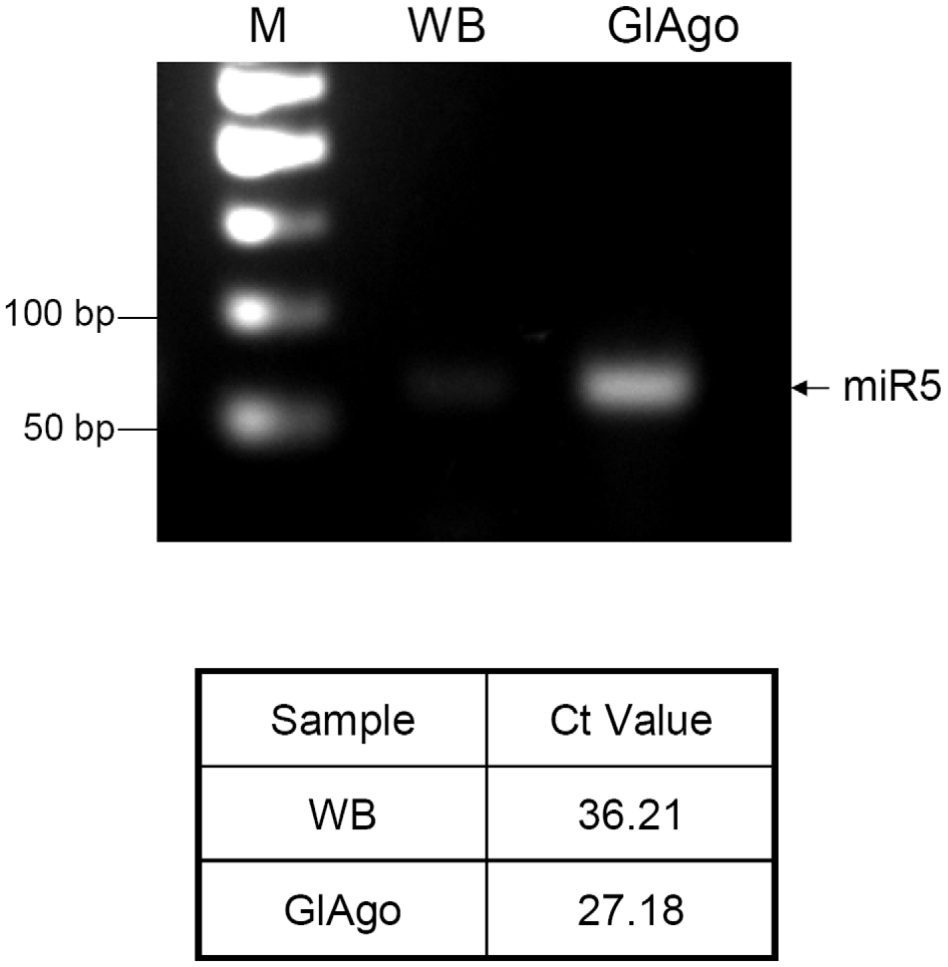
miR5 was co-immunoprecipitated with GlAgo. The ∼26 nt RNA band co-immunoprecipitated with 3×HA GlAgo [21] was analyzed by RT-qPCR using TaqMan probe specific for miR5. GlAgo: RNA template extracted from the immunoprecipitate of 3×HA GlAgo expressing cells. WB: RNA template extracted from the immunoprecipitate of control cells. The qPCR products were run on a 2% agarose gel. The specific band at ∼70 bp (26 nts plus the linker) showed miR5 co-immunoprecipitated with 3×HA GlAgo (*upper panel*). The Ct values between the two samples differed by about 9 cycles, suggesting that miR5 bound specifically to 3×HA GlAgo and became enriched in the immonoprecipitate (*lower panel*).

To further ascertain that miR5 is a functional miRNA that binds to GlAgo to form a potential RISC in *Giardia*, we immunoprecipitated GlAgo and examined if miR5 was associated with it. We have recently established a method of immunoprecipitating N-terminal 3×HA-tagged GlAgo from *Giardia* lysate and identified a single ∼26–30 nt RNA band associated with the protein that contained all the miRNAs we have identified thus far [21]. The miR5 level in this ∼26–30 nt RNA band was analyzed by RT-qPCR. The Ct value of the GlAgo pulled-down sample was about 9 cycles earlier than the Ct of the control sample (WB), suggesting that miR5 was significantly enriched in the GlAgo immunoprecipitate (Figure 5). miR5 is thus associated with GlAgo in *Giardia*, most likely in forming a RISC.

### miR5 exerts a target-site dependent inhibition of the expression of a reporter mRNA in *Giardia*

**Figure 6:**
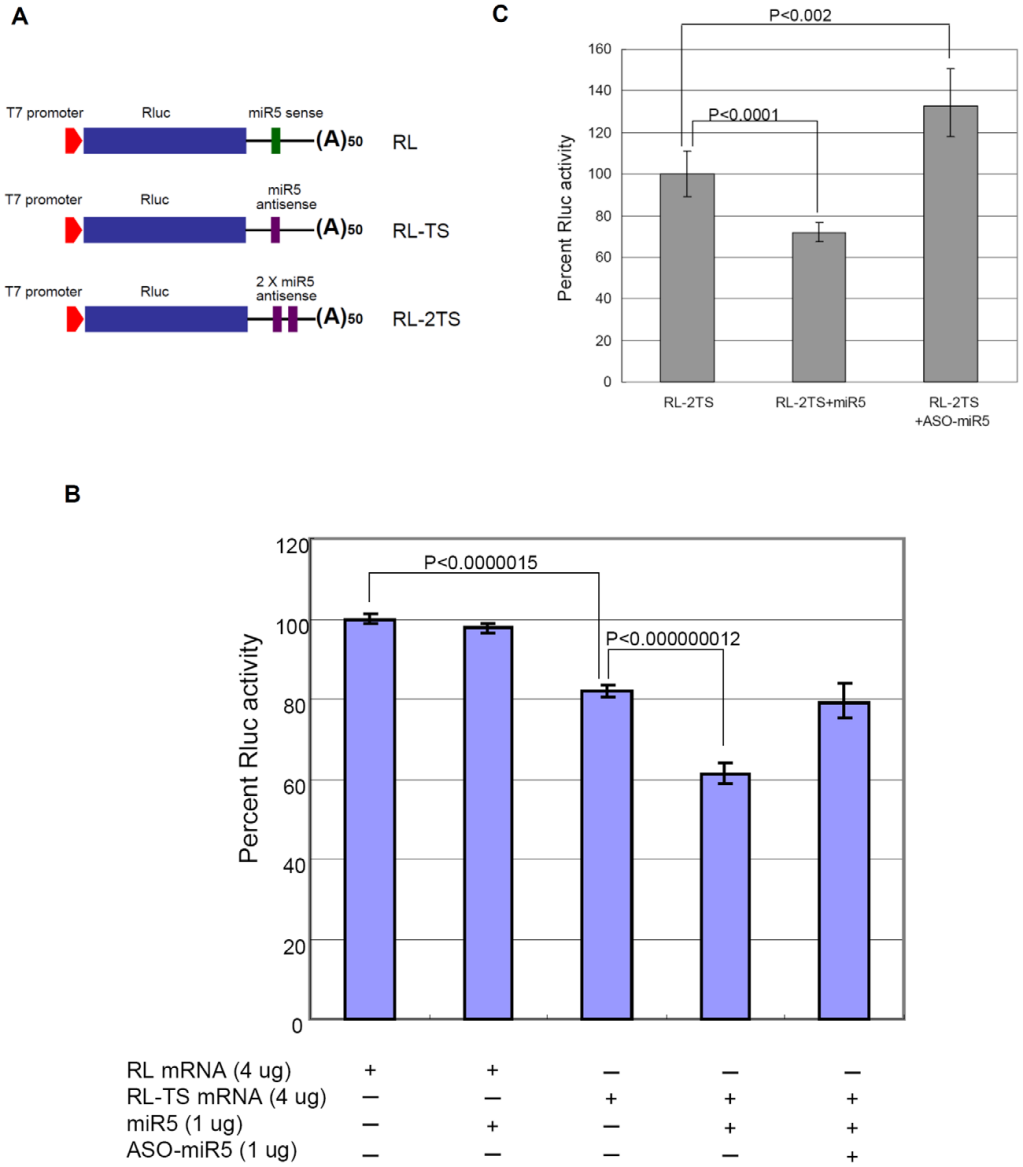
miR5 inhibits Rluc mRNA expression in *Giardia* by binding to the putative target site at 3′-UTR. (**A**) Diagram of the RL, RL-TS and RL-2TS reporter constructs. A copy of the miR5 sense sequence (green) and one (TS) or two (2TS) copies of the miR5 antisense sequence (purple) were each added to the 3′-UTR of *Renilla* Luciferase transcript as the negative control (RL) and transcripts carrying one and two potential miR5 target sites (RL-TS and RL-2TS), respectively. (**B**) Percentages of Luciferase activity expression in RL and RL-TS transcript transfected *Giardia* trophozoites. The Luciferase activity of RL was set at 100% and that of RL-TS was measured at 82%, suggesting action of endogenous miR5. The introduction of exogenous miR5 (1 µg) further represses RL-TS activity down to 61% without any detectable effect on RL expression. Inclusion of 1 µg of a 2′-O-methylated antisense of miR5 (ASO-miR5) with 1 µg of miR5 in the transfection recovers the Luciferase activity of RL-TS to the original 80%. The results and standard deviations were derived from three independent transfection experiments. The p-values indicated were calculated by two-tailed Student's *t*-test. (**C**) Effects of miR5 and ASO-miR5 on the expression of RL-2TS. The presence of double target sites shows a little more exogenous miR5 repression (∼30%) than that from the single target site shown in panel B. The introduction of ASO-miR5 without miR5 restores the expression of RL-2TS up to >130% apparently by inhibiting the endogenous miR5 function. The results and standard deviations were from three independent transfection experiments. The p-values indicated were calculated by two-tailed Student's *t*-test.

Most of the functional miRNAs reported to-date have been shown to bind to the 3′ UTR of their target mRNAs through base pairings and to exert an inhibition of translation of the mRNA [22]. Since miRNAs have usually multiple targets in a cell, the individual sites have typically reduced protein output by less than a half and often by less than a third from the action of a single miRNA [8]. To test the potential function of miR5 *in vivo*, the miR5 antisense sequence was incorporated into the 3′-UTR of a *Renilla* Luciferase (Rluc) cDNA, transcribed with T7 RNA Polymerase and capped *in vitro* (Figure 6A). The transcript RL-TS was co-transfected with synthetic miR5 into *Giardia* WB trophozoites and expressed at 37°C for 4 hrs. The cell lysate was then assayed for Rluc activity.

With the Luciferase expression from control mRNA with a miR5 sense sequence at the 3′UTR (RL) set at 100%, a co-transfection of synthetic miR5 with RL mRNA showed little effect on Luciferase expression (Figure 6B). A transfection with RL-TS mRNA alone, however, reached only 82% of Rluc expression, suggesting presence of endogenous miR5. A co-transfection of RL-TS with exogenous miR5 further reduced the Rluc expression to 61%. Apparently, the presence of endogenous miR5 in *Giardia* reduces the RL-TS expression by 18%, whereas the presence of additional synthetic miR5 further decreased the expression by a total of 39%. The inhibitory effect of the exogenously introduced miR5 is thus ∼21%.

To verify if the repressed Rluc expression requires hybridization between miR5 and the potential target site, a 2′-O-methylated antisense RNA sequence of miR5 (ASO-miR5) was synthesized [23]. The 2′-O-methylated antisense sequences are known to bind to the corresponding miRNAs in RISCs with extremely high affinity, effectively out-competing the target mRNA [23]. They represent a reliable tool in validating miRNA targets and studying the cellular functions of miRNAs. [23] When 1 µg of miR5 and 1 µg of ASO-miR5 were simultaneously introduced into the cells expressing RL-TS, the inhibition caused by exogenous miR5 was abolished and the Rluc activity increased back to the 80% level, equivalent to that induced by the endogenous miR5 alone (Figure 6B). Thus, the inhibitory effect of exogenous miR5 is most likely attributed to its binding to the potential target site (TS) and can be abolished by an equivalent amount of ASO-miR5. When the expression of an Rluc mRNA with 2 TSs separated by a 10 nt spacer in the 3′-UTR (RL-2TS) was assayed (Figure 6C), it was repressed by miR5 (1 µg) to 71%, a little more efficient than that observed with the single target site mRNA RL-TS (75%). When 1 µg of ASO-miR5 was introduced into the RL-2TS cells without introducing exogenous miR5, the Luciferase activity increased to >130% of the no ASO-miR5 control, suggesting that ASO-miR5 is also capable of inhibiting the function of endogenous miR5 (Figure 6C).

Levels of RL and RL-TS transcripts in the transfected *Giardia* were monitored by Northern blots (Figure S1). The results show little change in the levels of the mRNAs when the exogenous miR5 was introduced into the cells, indicating that the inhibited expression of Rluc by miR5 was not attributed to mRNA degradation, but more likely due to translation repression of the mRNA.

### miR5 loses its function in Argonaute-knockdown *Giardia* cells

**Figure 7:**
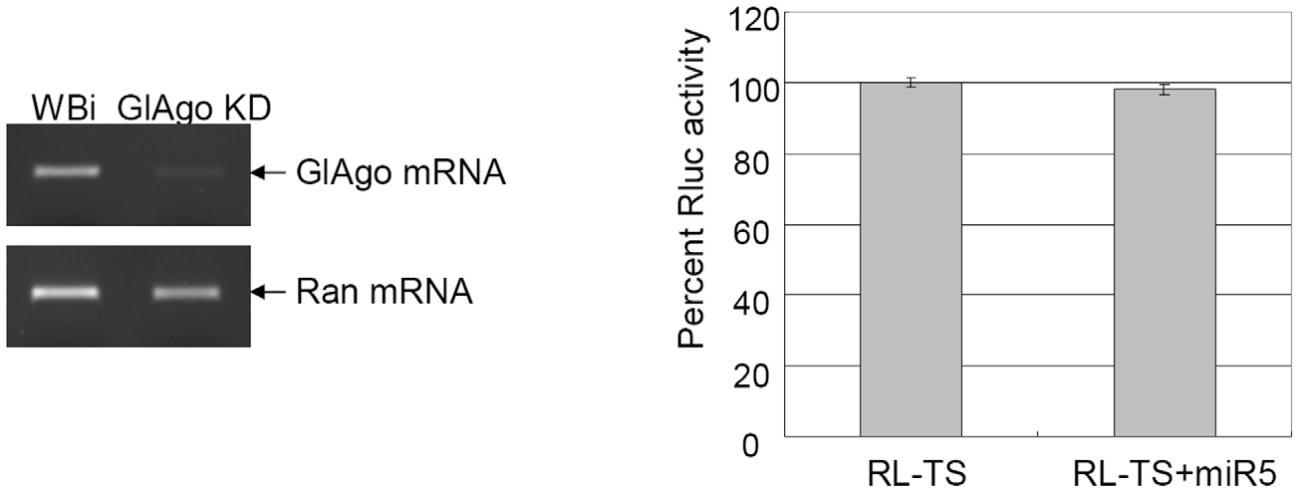
Effects of miR5 on RL-TS expression in GlAgo knockdown cells. Left panel: RT-PCR results showed that GlAgo mRNA was significantly reduced in the GlAgo knockdown *Giardia* cells (GlAgo KD). The Ran mRNA was monitored as a loading control. Right panel: Introduction of miR5 into the GlAgo KD cells had little effect on RL-TS expression.

The Argonaute protein is an integral component of RISC and plays an essential role in miRNA-mediated repression of mRNA translation [24]. To knock down *Giardia* Argonaute mRNA, a specific GlAgo-antisense-hammerhead ribozyme RNA was introduced into Giardiavirus-infected *Giardia* trophozoites as previously described [18]. The result indicated that the level of GlAgo mRNA was significantly reduced (Figure 7, right panel). The GlAgo knockdown cells were transfected with the RL-TS mRNA and the synthetic miR5, and the expression of Rluc was monitored. The results show that miR5 can no longer inhibit the expression of RL-TS in the GlAgo knock down cells and the Luciferase activity went back to the RL control level (Figure 7, left panel). Thus, GlAgo is required for the miR5-mediated repression of translation in *Giardia*.

### Search of potential binding sites for miR5 in *Giardia*

Current methods for predicting a target site of a miRNA have been diverse and require more room for improvement [25], [26]. However, three agreements appear to have emerged among them: (1) the need of conserved Watson-Crick pairing to the 5′ region of the miRNA centered on nucleotides 2–7 designated the seed-matched site; (2) Conserved pairing to the seed region can be sufficient on its own for predicting the targets; (3) an 8 nt match or multiple matches to the same miRNA increase the authenticity of the target. John et al. developed the miRanda algorithm to predict miRNA targets in different species. Three validation approaches (retrospective, statistical, and indirect experimental) showed that when the predicted targets are conserved and the gene contains more than one miRNA target sites or a single high-scoring site (the alignment score >110), genuine miRNA targets can be predicted at a reasonable accuracy [27]. We thus tried to predict the putative miR5 target sites in the *Giardia* genome using the miRanda program. Since *Giardia* mRNAs have relatively short 3′-UTRs and a growing number of mRNAs in other organisms have been found to be targeted by miRNAs within the open reading frames (ORFs) rather than the 3′-UTRs [28]–[30], segments of 100 nts with 50 nts upstream and 50 nts downstream from the stop codon of each ORF were extracted from the 4,889 ORFs in GiardiaDB [31], [32]. Twenty-one potential target sites for miR5 that bear a perfect complementation with nucleotides 2–7 in miR5 with a score threshold >120, and an energy threshold <−20 kcal/mol were selected (Table S1). Among them, 12 are located in the ORF region close to the stop codon, 9 are partially or totally in the 3′-UTR of the target mRNAs, whereas all 21 are upstream from the polyadenylation site (data not shown). For the 21 ORFs, 14 are hypothetical proteins and 7 are annotated proteins consisting of four kinases and three variant surface proteins (VSPs) (Table S1).

GlsR2 is a homologue of snoRNA U14 in yeast and vertebrates. The 3′ end, where miR5 is derived from, is the most conserved region. We went to deepBase [33], a platform for annotating and discovering small and long ncRNAs from deep-sequencing data, and found that there is a large number of small RNAs coming from the 3′ ends of human, mouse, chicken, *Ciona* and *Drosophila* U14 homologues, suggesting that miR5 may be conserved. We could not, however, find any homologue of the 21 potential *Giardia* mRNA targets of miR5 in other eukaryotes. Recent release of genome sequences and annotations of *Giardia intestinalis* GS [34] and *Giardia* E isolate P15 showed the presence of GlsR2 homologue in all three *Giardia* isolates. Comparative analysis of the 21 predicted mRNA targets among three isolates showed that 15 target sites were well conserved in at least one of the two other isolates, while the remaining 6 were found only in WB. Thus, a similar miR5-mediated regulation of gene expression could be functional among the three *Giardia* isolates.

**Figure 8:**
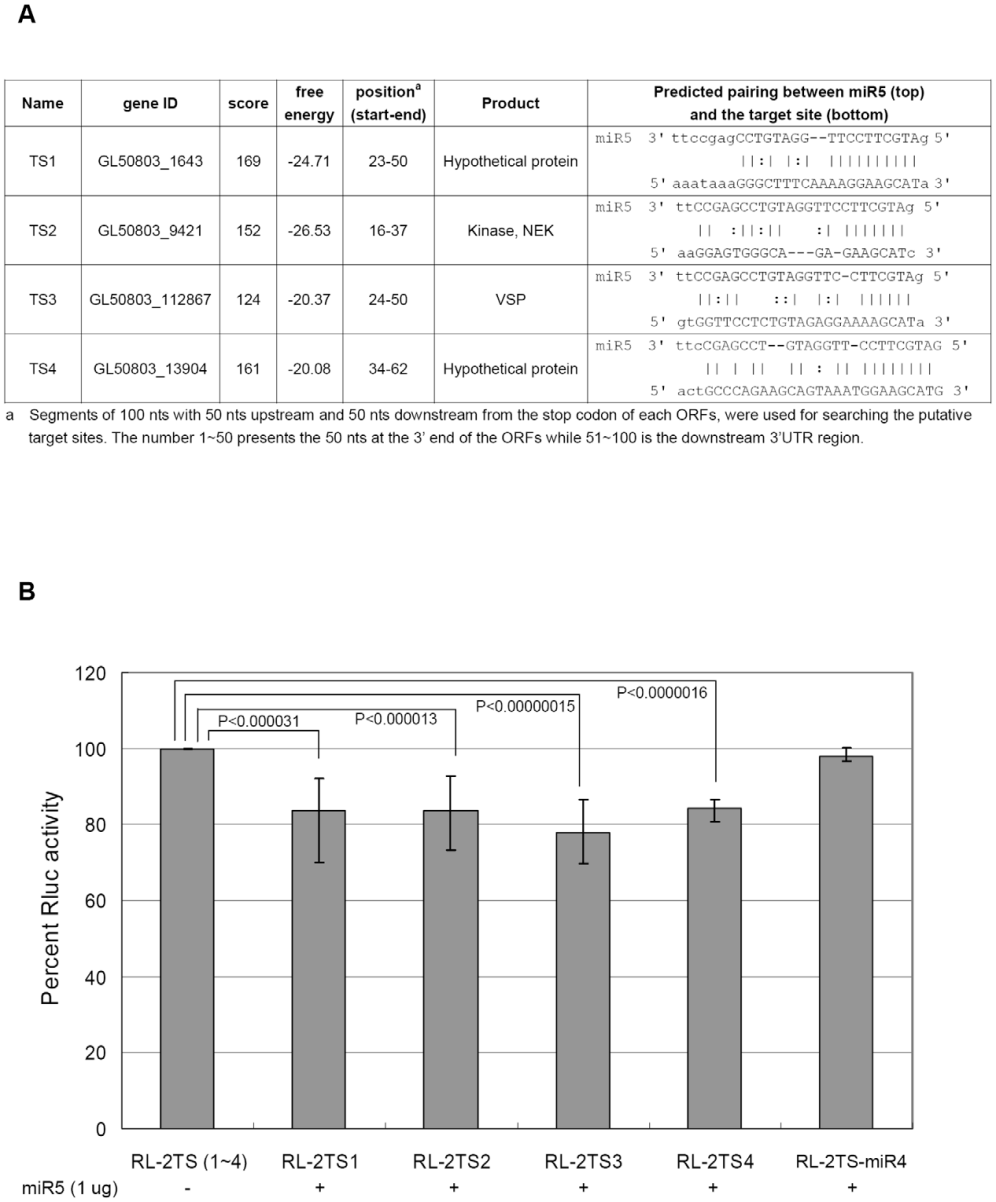
Experimental verification of potential target(s) for miR5. (**A**) The four genes containing potential miR5 target sites chosen for further test. (**B**) Two copies of each potential miR5 target site separated by a 10 nt spacer were cloned to the 3′-UTR of Rluc gene to generate four constructs: RL-2TS1, 2, 3 and 4. A Luciferase transcript carrying at the 3′-UTR a double miR4 target sites from another VSP (GL50803_36493) [21], unrelated to that of miR5, was included as a control (RL-2TS-miR4). The *in vitro* transcripts (4 µg) with or without 1 µg of synthetic miR5 were each introduced into *Giardia* via transfection and the subsequent Luciferase expression was monitored. The results and standard deviations were from three independent transfection experiments. The p-values indicated were calculated by two-tailed Student's *t*-test.

Four of the 21 potential target sites were selected for further study. The first site (TS1) is from a hypothetical protein gene, which has the highest score. The second site (TS2) is from one of the NEK kinase genes and has the lowest free energy. The third site (TS3) is from a VSP gene and has the lowest score, while the fourth site is from a hypothetical protein gene with the highest free energy (Figure 8A and Table S1). Duplicate copies of the four target sites were cloned into the 3′-UTR of Rluc mRNA (designated RL-2TS1, 2, 3 and 4) and the capped and *in vitro* transcribed RNA was transfected into *Giardia*. Luciferase assay showed that exogenous miR5 reduced the expression of the four mRNAs to 83.7%, 83.5%, 77.8% and 84.3% respectively, compared to the no miR5 control (Figure 8B). Another Rluc mRNA with two copies of the target site of a newly identified 26 nt miR4 at the 3′-UTR [21] was included as another control in the study. Like miR5, miR4 is playing a role in regulating VSP gene expression in *Giardia* [21]. This miR4 target site is from another VSP (GL50803_36493), which carries no target site for miR5. The result shows that miR5 has no detectable effect on the expression of this transcript carrying double target sites for miR4 (Figure 8B), suggesting that the miR5 repression of Rluc carrying TS1, 2, 3 or 4 is not a nonspecific phenomenon. The similar levels of repression from 15.7 to 22.2% among the four target sites suggest that the scores and free energies predicted by miRanda do not play a pivotal role in identifying miRNA target sites in *Giardia*. This could be attributed to the stringent criteria we used in predicting the target sites. All the conserved canonical 7–8 nt seed-matched sites enlisted in Table S1 could belong to the same family of authentic binding sites of miR5.

## Discussion

The presence of a miRNA pathway in *Giardia* has now been demonstrated by two examples of functional miRNAs, miR2 [18] and miR5, both of which are derived from snoRNAs. There is thus the probability that the snoRNAs may constitute a significant source of miRNAs in *Giarida*. Among the 16 putative *Giardia* box C/D snoRNAs identified thus far [19], 11 carry a single antisense element of rRNAs, whereas the other 5 have none, and could be classified as orphan snoRNAs. The corresponding rRNA targets postulated for the 11 box C/D snoRNAs were examined by primer extension [19]. Only GlsR1 showed an rRNA modification at the snoRNA-targeted site [19]. This suggests that most, if not all, of the 16 box C/D snoRNAs may not function as guides of 2′-O-methylation of rRNA but may perform some other functions, such as being the precursors of miRNAs in *Giardia*. GlsR2 is a homologue of snoRNA U14 in yeast and vertebrates but without the necessary A domain for the chaperon function of U14 [35]. Since the predicted site of methylation by GlsR2 was not verified by primer extension [19], it may also be an orphan snoRNA functioning as a precursor of miR5. However, it should not imply that snoRNAs constitute the only source of miRNAs in *Giardia*. Even in the apparent absence of Drosha/Pasha and Exportin5, there could be other potential precursor RNAs matured into miRNAs through digestion by Dicer and other yet un-identified enzyme(s) in *Giardia* [21].

The GlDcr protein has been found to localize to the cytoplasm of *Giardia* trophozoites [36], which raises the question on how a cytoplasmic enzyme can digest snoRNAs presumably localize to the nucleolus. In mammalian cells, box C/D snoRNAs are in the nucleolar snoRNP consisting of phosphorylated export adaptor PHAX, the cap binding complex Ran and the exportin CRM1, suggesting a cytoplasmic phase during the maturation of snoRNP [37]. This suggestion was supported by the observation that U8 and U22 snoRNAs injected into the cytoplasm were imported into the nuclei of *Xenopus* oocytes [38], and that U8 pre-snoRNPs had a distinct distribution in the nucleoplasm and cytoplasm with an association with both nuclear import and export factors during maturation [39]. snoRNAs thus appear to have a cytoplasmic phase during their maturation. Ran and CRM1 homologues have been identified in the *Giardia* genome database ([40], data unpublished). It is possible that the *Giardia* box C/D snoRNAs can be exported to the cytoplasm by the exportin CRM1 complex during their biogenesis and subject to processings to produce miRNAs in the cytoplasm. More recently, CRM1 was found to mediate nuclear-cytoplasmic shuttling of miRNAs and co-immunoprecipitate with Argonaute in mammalian cells [41]. It raises the interesting possibility that the same CRM1 complex could transport miRNAs and their snoRNA precursors, thus constituting a primitive pathway of post-transcriptional regulation in *Giardia*.

For the 7 annotated ORFs that carry potential target sites for miR5, three of them encode VSPs. These VSPs could be clustered in a subfamily according to their sequence similarity. They are different from the 22 VSP genes shown to carry the putative targets of miR2 [18], suggesting that, like miR2 [18], miR5 may also play a role in regulating the expression of VSPs in *Giardia*. Thus, a miRNA machinery may constitute an important mechanism of VSP gene regulation.

Most of the RNAs processed by Dicer result in a product with a 5′-monophosphate (5′-monoP) and a 3′-hydroxyl (3′-OH) structure which could be identified using the standard small RNA cloning techniques [13], [42]. However, certain small RNAs carry modified 5′ and/or 3′ termini. In *C. elegans*, secondary siRNAs contain a 5′-triphosphate group and are exclusively loaded into the secondary Argonautes (SAGOs) that can amplify the gene silencing effect [43]. These small RNAs with 5′-polyphosphate ends were also identified in the single-celled anaerobic eukaryote *Entamoeba histolytica* [44]. They were mainly mapped to the antisense genes and found associated with an *E. histolytica* Piwi-related protein [44]. In addition, miRNAs and siRNAs in plants, Piwi-interacting RNAs in animals and some miRNAs and siRNAs in *Drosophila* have 2′-O-methylation (2′OMe) on the 3′ terminal nucleotide [45]. This 3′-end modification can protect the small RNAs from 3′-end uridylation and 3′-to 5′-exonuclease-mediated degradation [46], [47]. However, the standard small RNA cloning method, which was used by us in identifying miR2 [18] and miR5 in *Giardia*, will not efficiently capture the small RNAs with a 5′-triphosphate structure [44]. The 2′-O-methylation at the 3′-end of RNA reduces also the efficiency of ligation by T4 RNA ligase by the standard cloning technique [48]. Recently, it was shown that small RNAs which can be co-immunoprecipitated with GlAgo have a 5′-monophosphate and a 3′-OH [21]. These facts, plus the experimental data that chemically synthesized miR2 and miR5 with 5′-monophosphates and 3′-OH's can be introduced into *Giardia* trophozoites and effectively repress the translation of mRNAs with the corresponding targeting sites at the 3′-UTRs provide a strong indication that these miRNAs have 5′-monophosphates and 3′-hydroxyls.

MicroRNAs constitute one of the more abundant classes of gene-regulatory molecules in animals [49]. Bioinformatic analysis and some high throughput experimental approaches showed that each miRNA has hundreds of evolutionarily conserved targets and a number of non-conserved targets [25], [50]–[52]. Recently, it was demonstrated that the majority of human genes (>60%) are under the control of miRNAs [49]. Moreover, Wu et al. showed that 28 miRNAs can modulate the expression of CDKN1A (p21^Cip1/Waf1^) by directly binding to its 3′-UTR [53], which confirmed that multiple miRNAs can target a single gene transcript. This suggests a complex network of interactions between miRNAs and mRNAs. We showed in this study that, experimentally, at least four genes and, bioinformatically, at least 17 additional genes in *Giardia* could be regulated by miR5. All the four experimentally demonstrated miR5 binding sites start in the ORF region and are located near or covering the stop codon. This might be a unique feature of miRNA-mediated gene regulation in *Giardia* due to the extremely short 3′-UTR of its mRNAs. Because the criteria we used for predicting targets are relatively stringent [52], we can expect that there are more miR5 targets in *Giardia*. Further experimental analysis of the remaining potential miR5 binding sites will eventually lead us to identify the complete spectrum of miR5 binding sites in *Giardia*.

The miRNA pathway in *Giardia* demonstrated by us is not well suited for defending the cells against the invading foreign dsRNA carried by the Giardiavirus. Those organisms equipped with the RNAi machinery such as *Trypanosoma brucei* [54], *Tetrahymena pyriformis* [55] or *Caenorhabiditis elegans* [56] have been known to be dsRNA virus free. miRNA-mediated gene repression is likely an antiquated and evolutionarily well conserved pathway of post-transcriptional gene regulation. The utilization of small hairpin structured RNAs, such as the snoRNAs, as the precursors of miRNAs suggests that the miRNA pathway could have evolved prior to the addition of Drosha/Pasha and Exportin5 into the pathway.

There was, however, one recent publication claiming the presence of RNAi in *Giardia* [36]. Although there was no actual RNAi experiment presented in the report, Prucca et al. showed that small pieces of VSP RNAs could be produced in *Giardia* extracts, and that multiple VSPs could be expressed on the cell surface when GlDcr or RNA-dependent RNA polymerase (RdRP) was partially knocked down using antisense RNA. These results led to the conclusion that VSP dsRNAs are synthesized by RdRP and processed by GlDcr for an RNAi mechanism of regulating VSP expression in *Giardia*. There was, however, no evidence showing that the small RNAs in *Giardia* extracts could be generated by GlDcr. In fact, there are so many RNases released in a *Giardia* extract, it is virtually not possible to claim a small RNA band being the product from Dicer action. There is also no slicer activity found in GlAgo, a key requirement for RNAi, and GlDcr is apparently incapable of digesting long dsRNAs (see above). There are thus still many uncertainties concerning the presence of a RNAi machinery in *Giardia*.

## Supporting Information

Figure S1 **Northern blot assays for RL and RL-TS mRNA from** ***Giardia*** **indicate that the presence of miR5 does not affect the level of RL-TS mRNA.** Quantification of the Northern results at the bottom was each derived from three independent transfections. The 16S rRNA was blotted as a loading control.
(TIF)

Table S1 **Predicted miR5 targeting sites in the** ***Giardia*** **genome using miRanda program.**
(DOC)
