# Supplementary figures and images for: Gene Regulation in Giardia lambia Involves a Putative MicroRNA Derived from a Small Nucleolar RNA
Source: PLoS Negl Trop Dis. 2011 Oct 18;5(10):e1338. doi: 10.1371/journal.pntd.0001338 (PMC3196473; doi:10.1371/journal.pntd.0001338)

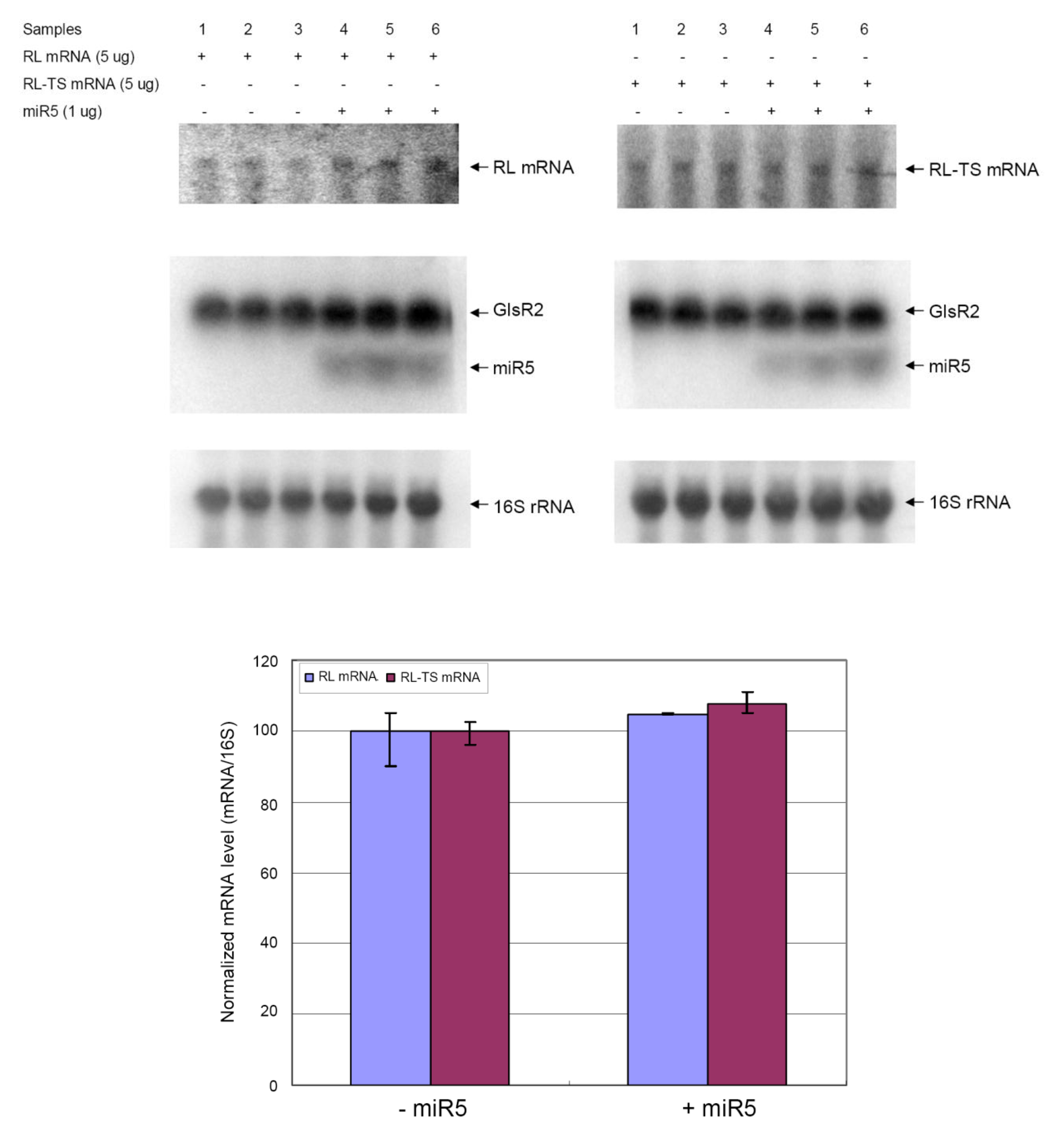

Supplement: Figure S1 — Northern blot assays for RL and RL-TS mRNA from Giardia indicate that the presence of miR5 does not affect the level of RL-TS mRNA. Quantification of the Northern results at the bottom was each derived from three independent transfections. The 16S rRNA was blotted as a loading control. (TIF) [file pntd.0001338.s001.tif]
