# Supplementary material for: Gene Regulation in Giardia lambia Involves a Putative MicroRNA Derived from a Small Nucleolar RNA
Source: PLoS Negl Trop Dis. 2011 Oct 18;5(10):e1338. doi: 10.1371/journal.pntd.0001338 (PMC3196473; doi:10.1371/journal.pntd.0001338)
Supplement: Table S1 — Predicted miR5 targeting sites in the Giardia genome using miRanda program. (DOC) [file pntd.0001338.s002.doc]

| **gene ID** | **score** | **free energy** | **position^a^ (start-end)** | **Product** | **Predicted pairing between**  **miR5 (top) and the target site (bottom)** | **Type^b^** |
| --- | --- | --- | --- | --- | --- | --- |
| GL50803_1643 | 169 | -24.71 | 23-50 | Hypothetical protein | miR5 3' ttccgagCCTGTAGG--TTCCTTCGTAg 5'  ||:| |:| ||||||||||  5' aaataaaGGGCTTTCAAAAGGAAGCATa 3' | 8mer |
| GL50803_21750 | 167 | -23.41 | 61-84 | Ribose-phosphate pyrophosphokinase | miR5 3' ttccgAGCCTGTAGGTTCCTTCGTAg 5'  |:|| | ||| |||||||||  5' tgttaTTGG-CTTCC-AGGAAGCATa 3' | 8mer |
| GL50803_6928 | 163 | -21.63 | 22-50 | Hypothetical protein | miR5 3' ttcCGAGCCTGTAGG---TTCCTTCGTAg 5'  || | | ||| ||||||||||  5' agaGCACTGCGCTCCAAAAAGGAAGCATa 3' | 8mer |
| GL50803_10063 | 155 | -21.17 | 47-76 | Hypothetical protein | miR5 3' ttccgagCCT-GTAGGTT---CCTTCGTAg 5'  ||| |:|:::: ||||||||  5' aatagaaGGACCGTTTGGCTTGGAAGCATa 3' | 8mer |
| GL50803_95549 | 150 | -21.69 | 6-40 | Kinase, NEK | miR5 3' ttCCGAGC--CTGTA-------GGTTCCTTCGTAg 5'  ||| || |:|| ||: |||||||  5' cgGGCGCGTCAATATAGAGGGGGCAGCGAAGCATa 3' | 8mer |
| GL50803_3370 | 168 | -21.26 | 49-76 | Hypothetical protein | miR5 3' ttCCG---AGCCTGT-AGGTTCCTTCGTAg 5'  ||| || ||| ||:|| |||||||  5' taGGCAGATC--ACAGTCTAACGAAGCATt 3' | 7mer-m8 |
| GL50803_29359 | 164 | -20.51 | 35-62 | Hypothetical protein | miR5 3' ttCCGAGC-C-TGTAGGTTCCTTCGTAg 5'  | ||:| | |||| |: |||||||  5' tcGACTTGAGTACATAGAGTGAAGCATg 3' | 7mer-m8 |
| GL50803_11692 | 163 | -22.43 | 57-80 | VSP, putative | miR5 3' ttcCGAGCCTGTAGGTTCCTTCGTAg 5'  | | ||| | ||| |||||||  5' gtaGATAGGAAAGCCA--GAAGCATc 3' | 7mer-m8 |
| GL50803_13904 | 161 | -20.08 | 34-62 | Hypothetical protein | miR5 3' ttcCGAGCCT--GTAGGTT-CCTTCGTAG 5'  || | || || : || ||||||||  5' actGCCCAGAAGCAGTAAATGGAAGCATG 3' | 7mer-m8 |
| GL50803_2555 | 160 | -23.12 | 56-83 | Hypothetical protein | miR5 3' ttCCGA-GCCT-GTAGGTTCCTTCGTAg 5'  || | :||| :| ::: ||||||||  5' tgGGATGTGGAGTAGTTGTGGAAGCATt 3' | 7mer-m8 |
| GL50803_8217 | 160 | -22.69 | 7-30 | Uridine kinase | miR5 3' ttCCGAGCCTGTAGGTTCCTTCGTAg 5'  | :|| ||||: ||||||||  5' gaGTTTCTTCCATCT--GGAAGCATc 3' | 7mer-m8 |
| GL50803_23858 | 160 | -20.83 | 18-40 | Hypothetical protein | miR5 3' ttcCGAGCCTGTAGGTTCCTTCGTAg 5'  ||||| ||::: |||||||  5' tgtGCTCG---ATTTGCTGAAGCATc 3' | 7mer-m8 |
| GL50803_9421 | 152 | -26.53 | 16-37 | Kinase, NEK | miR5 3' ttCCGAGCCTGTAGGTTCCTTCGTAg 5'  || :||:|| :| |||||||  5' aaGGAGTGGGCA---GA-GAAGCATc 3' | 7mer-m8 |
| GL50803_26439 | 152 | -20.77 | 43-72 | Hypothetical protein | miR5 3' ttccGAGCCTGTAGGTTC----CTTCGTAg 5'  :|: |::: || || |||||||  5' atccTTTAGGTGCCCCAGAGATGAAGCATc 3' | 7mer-m8 |
| GL50803_117311 | 152 | -20.14 | 15-41 | Hypothetical protein | miR5 3' ttCCGAG---CCTGTAGGTTCCTTCGTAg 5'  | ||: |||| | | |||||||  5' atGCCTTACAGGAC--CGATAGAAGCATc 3' | 7mer-m8 |
| GL50803_117312 | 152 | -20.14 | 15-41 | Hypothetical protein | miR5 3' ttCCGAG---CCTGTAGGTTCCTTCGTAg 5'  | ||: |||| | | |||||||  5' atGCCTTACAGGAC--CGATAGAAGCATc 3' | 7mer-m8 |
| GL50803_98638 | 136 | -22.92 | 17-50 | Hypothetical protein | miR5 3' ttCCGAG---CCTGTAGGTTC-----CTTCGTAG 5'  | ||| | ||| ||||| ||||||  5' caGCCTCAGTGCACAGCCAAGGTTGCCAAGCATA 3' | 7mer-A1 |
| GL50803_14937 | 134 | -21.05 | 51-81 | Hypothetical protein | miR5 3' ttccgaGCCT----GTAGGTTCC-TTCGTAG 5'  |||| :||| | || ||||||  5' ggtggaCGGAAAACTATCGATGGTAAGCATA 3' | 7mer-A1 |
| GL50803_6459 | 133 | -22.01 | 8-43 | Hypothetical protein | miR5 3' ttcCG-AGCCTGTAG-GT--------TCCTTCGTAg 5'  || |||||| || |: || ||||||  5' gccGCGTCGGACCTCACGGTTGGGTCAGAAAGCATa 3' | 7mer-A1 |
| GL50803_137681 | 125 | -21.66 | 17-50 | VSP | miR5 3' ttcCGA--GCC-TGTAGG---TTCC--TTCGTAg 5'  ||| :|| ||||: :||| ||||||  5' tctGCTGGTGGTTCATCTGTAGAGGAAAAGCATa 3' | 7mer-A1 |
| GL50803_112867 | 124 | -20.37 | 24-50 | VSP | miR5 3' ttCCGAGCCTGTAGGTTC-CTTCGTAg 5'  ||:|| ::| |:| ||||||  5' gtGGTTCCTCTGTAGAGGAAAAGCATa 3' | 7mer-A1 |

a Segments of 100 nts with 50 nts upstream and 50 nts downstream from the stop codon of each ORFs, were used for searching the putative target sites. The number 1~50 presents the 50 nts at the 3’ end of the ORFs while 51~100 is the downstream 3’UTR region.

b The types of target site are annotated according to reference 44. 8mer means “seed” (nucleotides 2-7 of miRNA) match, plus an A residue across the first nucleotide of miRNA, and a match at the 8th nucleotide of miRNA. 7mer-m8 means “seed” match, and an A residue across the first nucleotide of miRNA. 7mer-A1 means “seed” match, and a match at the 8th nucleotide of miRNA.
